# Ultrasound Core Laboratory for the Household Air Pollution Intervention Network Trial: Standardized Training and Image Management for Field Studies Using Portable Ultrasound in Fetal, Lung, and Vascular Evaluations

**DOI:** 10.1016/j.ultrasmedbio.2021.02.015

**Published:** 2021-06

**Authors:** Víctor G. Dávila-Román, Ashley K. Toenjes, Rachel M. Meyers, Pattie M. Lenzen, Suzanne M. Simkovich, Phabiola Herrera, Elizabeth Fung, Aris T. Papageorghiou, Rachel Craik, John P. McCracken, Lisa M. Thompson, Kalpana Balakrishnan, Ghislaine Rosa, Jennifer Peel, Thomas F. Clasen, Shakir Hossen, William Checkley, Lisa de las Fuentes, for the HAPIN Investigators

**Affiliations:** ⁎ Cardiovascular Imaging and Clinical Research Core Laboratory, Cardiovascular Division, Department of Medicine, Washington University in St. Louis, Missouri, USA; † Division of Pulmonary and Critical Care, Johns Hopkins University School of Medicine, Baltimore, Maryland, USA; ‡ Center for Global Non-Communicable Disease Research and Training, School of Medicine, Johns Hopkins University, Baltimore, Maryland, USA; § Nuffield Department of Women's and Reproductive Health, University of Oxford, Oxford, UK; ¶ Centre for Health Studies, Universidad del Valle de Guatemala, Guatemala City, Guatemala; ║ Nell Hodgson Woodruff School of Nursing, Emory University, Atlanta, Georgia, USA; # ICMR Center for Advanced Research on Air Quality, Climate and Health, Department of Environmental Health Engineering, Sri Ramachandra Institute of Higher Education and Research, Chennai, India; ⁎⁎ Department of Disease Control, London School of Hygiene and Tropical Medicine, London, UK; †† Department of Environmental and Radiological Health Sciences, Colorado State University, Fort Collins, Colorado, USA; ‡‡ Department of Environmental Health, Rollins School of Public Health, Emory University, Atlanta, Georgia; USA

**Keywords:** Ultrasound, Core Laboratory, Sonographer, CIMT, BART, Lung ultrasound, Fetal ultrasound, Education, Multidisciplinary, Competency, Quality control

## Abstract

Ultrasound Core Laboratories (UCL) are used in multicenter trials to assess imaging biomarkers to define robust phenotypes, to reduce imaging variability and to allow blinded independent review with the purpose of optimizing endpoint measurement precision. The Household Air Pollution Intervention Network, a multicountry randomized controlled trial (Guatemala, Peru, India and Rwanda), evaluates the effects of reducing household air pollution on health outcomes. Field studies using portable ultrasound evaluate fetal, lung and vascular imaging endpoints. The objective of this report is to describe administrative methods and training of a centralized clinical research UCL. A comprehensive administrative protocol and training curriculum included standard operating procedures, didactics, practical scanning and written/practical assessments of general ultrasound principles and specific imaging protocols. After initial online training, 18 sonographers (three or four per country and five from the UCL) participated in a 2 wk on-site training program. Written and practical testing evaluated ultrasound topic knowledge and scanning skills, and surveys evaluated the overall course. The UCL developed comprehensive standard operating procedures for image acquisition with a portable ultrasound system, digital image upload to cloud-based storage, off-line analysis and quality control. Pre- and post-training tests showed significant improvements (fetal ultrasound: 71% ± 13% vs. 93% ± 7%, *p* < 0.0001; vascular lung ultrasound: 60% ± 8% vs. 84% ± 10%, *p* < 0.0001). Qualitative and quantitative feedback showed high satisfaction with training (mean, 4.9 ± 0.1; scale: 1 = worst, 5 = best). The UCL oversees all stages: training, standardization, performance monitoring, image quality control and consistency of measurements. Sonographers who failed to meet minimum allowable performance were identified for retraining. In conclusion, a UCL was established to ensure accurate and reproducible ultrasound measurements in clinical research. Standardized operating procedures and training are aimed at reducing variability and enhancing measurement precision from study sites, representing a model for use of portable digital ultrasound for multicenter field studies.

## Introduction

Globally, nearly three billion people rely on solid biomass fuels for cooking and heating. The resulting household air pollution is a leading environmental risk factor, accounting for an estimated 1.6 million premature deaths annually (Bonjour et al. 2013; GBD 2017 Risk Factor Collaborators 2018). The Household Air Pollution Intervention Network (HAPIN) trial is an ongoing randomized controlled trial of an intervention involving liquefied-petroleum-gas stoves and fuel distribution in 3200 households in four low- and middle-income countries: India, Guatemala, Peru and Rwanda (Clasen et al. 2020). The HAPIN study has recruited 800 pregnant women in each country, with households randomly assigned to receive liquefied-petroleum-gas stoves versus continued use of a biomass-burning stove; older adult women from the same households were also enrolled. Primary health outcomes are birth weight, incidence of severe pneumonia in the children, stunted growth in the children and blood pressure in the older adult women. Field portable ultrasound is used to evaluate secondary ultrasound imaging biomarker endpoints, which include fetal, lung and vascular ultrasound (carotid intima media thickness [CIMT] and brachial artery reactivity testing [BART]).

Ultrasound is a powerful imaging technique used clinically by multiple medical specialties. For research studies, ultrasound biomarkers are used to define robust phenotypes. An Ultrasound Core Laboratory (UCL) is frequently used in multicenter trials to reduce image variability and enhance measurement precision of imaging endpoints, and to allow blinded independent review (Gottdiener et al. 2004; Douglas et al. 2009; Gillam et al. 2017). For the HAPIN trial, a modern portable ultrasound system was used to obtain high-resolution images for evaluation of longitudinal imaging biomarker endpoints. Sonographers underwent additional formal ultrasound training in fetal, lung and vascular ultrasound. Image acquisition occurred in the field at the homes of study participants or at area health clinics. The aims of this report are to describe the administrative and training roles played by the UCL in standardizing the training and evaluation of sonographers and in acquiring and interpreting ultrasound studies. HAPIN sonographers were cross-trained to measure all four types of ultrasound imaging biomarkers (*i.e.,* fetal, lung, CIMT and BART). The UCL ensured accurate and reproducible ultrasound measurements to minimize systematic errors associated with pooling data from different study sites, allowing robust analysis when the imaging data set is merged with other data sources (*e.g.,* exposure, clinical). Thus, meticulous standardization and continuous monitoring for adherence to high-quality ultrasound imaging and measurement protocols is considered an essential function of the UCL, as described in this report.

## Materials and Methods

### Description of the UCL

The UCL is charged with providing qualified personnel, equipment, facilities, standardized protocols (*i.e.,* standard operating procedures [SOPs]), training materials, data collection forms, database development and analysis strategies to perform the tasks associated with the imaging endpoints for the HAPIN study. These activities are performed in coordination with the HAPIN study Clinical and Imaging Core, the Steering Committee, the International Research Centers (IRCs) and the Data Management Center. The UCL is located at Washington University in St. Louis and is operated by an academic imaging core laboratory associated with a not-for-profit institution with a long track record of performing core laboratory functions for many multicenter trials funded by the National Institutes of Health and for clinical studies with data submitted to the U.S. Food and Drug Administration (http://asecho.org/what-we-do/research-resources/academic-imagingcore-labs/).

The trial is registered on ClinicalTrials.gov (NCT02944682; Checkley, Clasen, Peel). The study protocol has been reviewed and approved by institutional review boards or ethics committees at Emory University (00089799), Johns Hopkins University (00007403), the Sri Ramachandra Institute of Higher Education and Research (IEC-N1/16/JUL/54/49) the Indian Council of Medical Research–Health Ministry Screening Committee (5/8/4-30/(Env)/Indo-US/2016-NCD-I), Universidad del Valle de Guatemala (146-08-2016/11-2016), the Guatemalan Ministry of Health National Ethics Committee (11-2016), A.B. PRISMA, the London School of Hygiene and Tropical Medicine (11664-5), the Rwandan National Ethics Committee (No.357/RNEC/2018), and Washington University in St. Louis (201611159). Results are disseminated to the appropriate stakeholders through presentations, conferences and peer-reviewed journals.

### Portable ultrasound system

All sites were provided the same ultrasound system (Sonosite Edge, Fujifilm Sonosite Inc., Bothell, WA, USA) to achieve image uniformity across sites. Each ultrasound system is equipped with three transducers (phased array, curvilinear and linear) and loaded with software for DICOM imaging and PACS network interface, calculation packages, additional batteries and charger (Thomas et al. 2005). When training was completed, most sonographers returned to their home countries with their ultrasound systems. Owing to regulatory requirements, ultrasound systems were shipped to India.

### Sonographer training

Remote online and in-person on-site ultrasound training of local sonographers from the four IRCs consisted of five phases:

• Phase 1. Identification of qualified site sonographers (three or four per IRC), recruited by local study staff based on prior sonography and/or health care experience.
• Phase 2. Introductory webinars by the UCL directors to review basic ultrasound principles, epidemiology and imaging protocols. These were held in the wk preceding the face-to-face hands-on training (Table 1).

**Table 1:** Lecture topics for in-person ultrasound training seminar

Area	Topics
General ultrasound	Ultrasound physics
	Transducer characteristics
	Image acquisition
	Echogenicity and ultrasound artifacts
	Introduction to the Sonosite ultrasound system and functionality of knobs
Fetal ultrasound	Fetal anatomy
	Fetal number
	Viability and heart rate
	Pregnancy dating
	Biometry
	Presentation and placentation
	Amniotic fluid
	Fetal ultrasound cases
Lung and pleura ultrasound	Lung and pleura anatomy
	Normal lung ultrasound
	Lung and pleura ultrasound for evaluation of pneumonia
	Pneumonia lung ultrasound cases
Carotid ultrasound	Epidemiology of carotid artery atherosclerosis
	General blood vessel anatomy
	Carotid artery anatomy and ultrasound imaging planes
	Image acquisition and measurement of carotid intima media thickness
	Identification and characterization of clinically significant atherosclerotic plaque
	Carotid artery ultrasound cases
Brachial artery reactivity testing	Physiology of brachial artery flow-mediated dilation
	Characteristics of normal and abnormal flow-mediated response
	Protocol and technique for use of blood pressure equipment to establish forearm ischemia
	Brachial artery reactivity testing image acquisition before and after ischemia
	Brachial artery reactivity testing cases
Household Air Pollution Intervention Network trial standard operating procedures	Ultrasound quality control programs
	Ultrasound upload for cloud storage (Trice Imaging)
	Completion of Trice and REDCap data collection forms
• Phase 3. Face-to-face ultrasound didactic and supervised hands-on training (2 wk held during the formative stage of the trial in 2017) at Washington University in St. Louis.
• Phase 4. Sonographer certification, performed after in-person training was completed and sonographers returned to their respective countries. This consisted of acquisition and submission of studies until 25 cases meeting all quality metrics were accumulated per image type for individual sonographer certification.
• Phase 5. Continuous training and re-training to accommodate staffing changes and provide remote online and/or as-needed on-site “refreshers” for sonographers not meeting quality control (QC) metrics, and to train additional sonographers as needed.

### Program faculty and support staff

Expert program faculty and staff included UCL physician-investigators and highly trained sonographers with extensive experience in fetal, lung and vascular ultrasound from maternal-fetal, pulmonary, cardiology and radiology specialties from Washington University in St. Louis, Missouri, USA; Johns Hopkins University in Baltimore, Maryland, USA; and the University of Oxford in Oxford, UK. The UCL physician-investigators provide relevant expertise in interpreting and contextualizing the broad-ranging imaging biomarkers and assist with joint analysis of imaging data with clinical, exposure and other study phenotypes. The UCL team fosters multidisciplinary collaboration and scientific creativity and provides consultation with other HAPIN investigators to discuss ongoing projects, opportunities and developments in imaging biomarker research, novel imaging techniques, ideas for future proposals and training of global health investigators.

### Description of didactic and hands-on ultrasound training program

A training curriculum incorporating didactic and practical scanning sessions for all ultrasound assessments was developed during the formative phase of the trial. Initial introductory webinars were held with site sonographers to review basic ultrasound principles, epidemiology and imaging protocols (written in both English and Spanish) in the wk preceding the in-person training. The comprehensive in-person face-to-face ultrasound curriculum was taught over a 2 wk period by the UCL team. The research ultrasound framework was designed to teach high-quality ultrasound techniques specifically focusing on the relevant imaging biomarker endpoints of the study. This framework integrated practical, supervised ultrasound training sessions that focused on four components: overall HAPIN trial research aims and hypotheses; imaging protocols; image acquisition, interpretation and submission to UCL; and completion of case-report forms. Sonographers had extensive hands-on scanning opportunities that included structured simulation labs and scanning sessions with live participants using the portable ultrasound systems. Ultrasound lectures and laboratories included the topics outlined in Table 1. Lectures were complemented daily by 4 h hands-on workshops that included both simulations and real-time imaging of healthy participants for performance of lung ultrasound, CIMT and BART and pregnant volunteers for fetal ultrasound based on previous experiences and research (de Las Fuentes et al. 2008, 2009; Nguyen-Thanh and Benzaquen 2009; Painschab et al. 2013; Papageorghiou et al. 2014a, 2014b; Reddy et al. 2014; Ellington et al. 2017; Lin et al. 2017; Goodman et al. 2019; McGill et al. 2019; Minardi et al. 2019; Simkovich et al. 2020).

The 2 wk in-person practical sessions were supplemented by use of an interactive obstetric multimedia training package using still and dynamic ultrasound imagery, audio narration, computer graphic imagery and animation, along with a transducer simulator, to allow review of key ultrasound pathology (SonoSim, Santa Monica, CA, USA). Step-by-step ultrasound imaging guides (single-page “knobology” guides) were developed and made available to each sonographer. These guides provided detailed instructions on transducers, machine settings, patient positioning, initial transducer placement and strategies to identify the anatomic structures of importance to the study. Card decks of representative images and diagrams of sonographic anatomy were issued to provide easy reference.

### Ultrasound image acquisition protocols

SOPs, imaging protocols and data collection forms were developed to standardize the acquisition and measurement of the ultrasound biomarkers (fetal, lung, CIMT and BART). Comprehensive ultrasound imaging protocols were developed with input from experts in each respective field of fetal, lung and vascular ultrasound using standardized and well-accepted methods. Sonographer training was focused on obtaining high-quality images in compliance with the ultrasound imaging protocols.

### Sonographer evaluation

Sonographers were evaluated during class participation and by written and practical hands-on tests. Written ultrasound material was assessed by two separate ultrasound-specific 25-question multiple-choice tests administered before and after the ultrasound training course. The test was based on the lecture content and included recognition of ultrasound concepts and identification of relevant ultrasound anatomic structures, whereas hands-on training focused on proper technique and recognition of relevant anatomic structures. An end-of-course practical assessment was administered in which sonographer trainees were asked to complete a full research-grade ultrasound study for evaluation by an expert proctor, with written feedback evaluations provided after completion.

### Continued sonographer training and certification

After the 2 wk ultrasound training program at Washington University in St. Louis, UCL expert sonographers traveled to individual IRCs to continue additional didactic and hands-on training in the conduct and interpretation of the ultrasound studies and to assist with individual sonographer certifications. Site sonographers were required to independently and successfully perform and upload 25 full ultrasound studies per imaging modality into a secure, cloud-based digital image management server . UCL members assessed image quality for interpretation and off-line measurements. For lung ultrasound only, site sonographers were required to perform a pre-specified number of ultrasound studies under direct supervision and then correctly interpret >85% of standardized ultrasound videos. Full certification was awarded after sonographers properly performed, interpreted and submitted 25 ultrasound studies meeting all QC metrics for each fetal, lung, CIMT and BART study.

### UCL imaging database

The UCL developed and maintains a secure, web-based, password-protected database to coordinate collection, processing and storage of all de-identified digital ultrasound images for all imaging biomarkers. The image storage system facilitates data analysis and retrieval and optimizes collaboration between projects and investigators. In coordination with the data coordinating center, the UCL ultrasound imaging biomarker database is shared with HAPIN investigators for analysis.

### Cloud-based digital image storage and off-line ultrasound image analysis management system

Image storage, management and interpretation are handled with a cloud-based, secure digital image repository (Trice Imaging Inc., Del Mar, CA, USA) that allows for image interpretation as needed. This system allows for images to be securely uploaded either directly from the portable ultrasound systems or from a local computer or image server (Table 2). After ultrasound images are uploaded, they are downloaded to the Washington University, Oxford University and Johns Hopkins University servers for subsequent storage, evaluation and blind interpretation by UCL sonographers. Trice and REDCap case report forms are completed online and linked to each individual ultrasound image set.

**Table 2:** Tools for image interpretation in the Household Air Pollution Intervention Network trial using Trice Imaging

Administrative	
Assignment of tasks	Tasks assigned to any registered user
Task notification	Sonographer notified of number of assigned tasks by a push notification button in the main control panel
Track tasks, view task status	Task status changes from “unread” to “started” when a sonographer starts reading an assigned study, and the sonographer marks as “completed” when finished. The moderator can see the current status of a task
Report completion	A form can be completed to record the interpretation of each scan
Blinding	Original images are preserved to allow for assignment of the images to different readers. Each reader can be unbiased (blinded) to the ultrasound images, measurements, notes and interpretation (report completion) of another reader
Separate folder	Operator can create separate folder for training images and main trial images, and access to these folders can be controlled to assign sonographers to review studies in an unbiased (blinded) fashion
Technical	
Still-image capture	Still images can be captured and saved from any scan uploaded into the platform
Measurement tools	Images can be measured in all directions; multiple measurements possible on any image. Labels are available to define the measurement made. Measurements are made to 1 mm
Video speed	Video can be stopped or played forward/backward by individual frames; play speed can be adjusted
Notes	Sonographers and readers can post notes accessible to other team members, such as to request clarification or provide comment regarding scan quality or diagnosis

The workflow for image interpretation, while individualized for the different ultrasound modalities, provides an integrated approach to meet two overarching objectives: IRC site sonographer interpretation of ultrasound images and continuous sonographer evaluations and data reports to investigators and stakeholders. The first step involves the IRC site sonographer uploading the ultrasound images to the Trice cloud-based system and completing the ultrasound case report form. The site-sonographer evaluation, in addition to serving the important purpose of ensuring that sonographers obtain high-quality images to allow adequate measurements for study data, is officially recorded and undergoes quality control. For CIMT studies, images are analyzed using Sonosite IMT edge-detection software, which allows fast and efficient UCL QC evaluation, finalization of measurements and entry to the imaging database.

From a managerial standpoint, the platform offers a dashboard that allows assignment of tasks to sonographers or experts who are performing interpretation, QC checks and case reports. In addition, unbiased (blinded) interpretation of ultrasound images can be completed by one or more individuals. The dashboard allows for the coordinator to be notified when a task is complete (such as an interpretation), to allow for tracking. This platform was chosen for its ease of use, image accessibility and affordability.

### Ultrasound image QC

The UCL oversees all stages of QC, including training, standardization, monitoring of sonographers and continuous assessment of image quality and consistency of measurements. The UCL's rigorous ongoing QC program is designed to evaluate and ensure adherence to the ultrasound imaging SOPs and provide feedback to each IRC sonographer. After sonographers perform measurements of fetal, lung and vascular ultrasound images, the UCL performs unbiased (blinded) independent QC on all ultrasound images at our centralized facility to ensure 100% adherence to the standard imaging study protocol. The QC program has three components: evaluate and report the technical quality of the ultrasound images; identify deviations from the imaging protocol (*i.e.,* non-adherence to imaging protocols or suboptimal image quality precluding evaluation or measurements) and provide re-training for site sonographers; and determine inter- and intra-observer reproducibility of analysis. QC metrics have been developed for each ultrasound imaging biomarker; failure to meet a minimum threshold prompts sonographer re-training, which consists of additional didactic training with the lead sonographer and acquisition and submission of an additional 5–10 certification studies for UCL review. A unique QC program has been developed for each ultrasound imaging biomarker (*i.e.,* fetal, lung, vascular) to address particular issues with each modality.

### Statistical analysis

Analyses of survey data are presented as mean ± standard deviation. Paired *t*-tests were used to compare pre- versus post-test results.

## Results

### Performance during initial training

For the initial sonographer training, assessments took place during the 2 wk in-person on-site training. Assessments showed significant score improvements from before to after ultrasound training, as follows—fetal ultrasound: 71% ± 13% versus 93% ± 7% (*p* < 0.0001); vascular/lung ultrasound: 60% ± 8% versus 84% ± 10% (*p* < 0.0001). All but one sonographer passed all ultrasound components, including ultrasound-specific tests and practical hands-on examinations. Additional training was recommended for individual sonographers; this training was completed during phase 4 of the ultrasound training plan.

### Survey outcomes

Ultrasound training evaluation from all sonographers (100% response rate) rated the program very favorably: mean score, 4.9 ± 0.1 (scale: 1 = worst to 5 = best). Qualitative written feedback also showed high satisfaction with training and confidence with the newly acquired ultrasound skills.

## Discussion

This report describes the Ultrasound Core Laboratory used in the HAPIN trial, a multicountry randomized controlled trial conducted in Guatemala, Peru, India and Rwanda. The HAPIN trial evaluates the effects of a liquefied-petroleum-gas stove intervention to decrease household air pollution and improve health outcomes. Comprehensive UCL protocols for the trial, including collection, interpretation and quantification of ultrasound data, are described in this article. Specific UCL functions include developing ultrasound imaging protocols, training sonographers for image acquisition, overseeing image acquisition, analyzing ultrasound data, administering the QC program, managing images and data (including digital file transfer, upload and storage), measuring images and preparing and submitting data reports and manuscripts. The ultrasound QC program oversees the process, including initial training, standardization, performance monitoring of sonographers and continuous assessment of image quality and consistency of measurements. The comprehensive protocol for fetal, lung and vascular (*i.e.,* CIMT, BART) ultrasound included a training curriculum that incorporated didactics, practical scanning and written/practical assessments which showed that participating sonographers performed well. Furthermore, a long-term QC program ensures that sonographers are monitored and re-trained as needed.

Ultrasound is commonly available in many health care settings in high-income countries, including as point-of-care testing in emergency departments, and it is becoming increasingly more widespread in low-resource settings. Ultrasound systems have been widely available worldwide for clinical care for almost 40 y, and offer a number of advantages including portable, non-invasive imaging without ionizing radiation, wide acceptability by study participants, ease of use after appropriate training and relatively low cost. Furthermore, whereas ultrasound already plays important roles in a large and diverse number of medical subspecialties, the widespread availability of affordable portable ultrasound systems, digital archiving and the ability to perform off-line measurements further expand its reach in both clinical and research contexts.

In clinical research settings, ultrasound is an invaluable and powerful tool for evaluating important imaging biomarkers for exquisite phenotyping. Here we report on the development of a robust UCL for the administration and training of sonographers to measure the ultrasound imaging biomarkers (*i.e.,* fetal, lung, CIMT and BART) required for the HAPIN study using a portable common ultrasound system.

Through the UCL, and under the supervision of well-trained physicians and sonographers with extensive expertise in their respective ultrasound imaging tests and in conducting clinical trials, we developed SOPs, training protocols, site sonographer training and interpretation of ultrasound data, with unbiased (blinded) central interpretation of the images under the direction of ultrasound-trained expert physicians-investigators. QC measures to assess and minimize variability in acquisition and reading were implemented.

### Limitations and challenges of the study, and proposed solutions

The use of portable ultrasound systems with limited resources, particularly in low- and -middle-income countries, presents unique limitations and challenges that require recognition and backup plans to address and resolve. First, sonographer training could be inadequate for performance of the proposed studies. While this is possible, the extensive online and in-person training demonstrated that the sonographers both had knowledge of ultrasound concepts and were able to obtain the required images. Through the follow-up on-site training, continuous monitoring and QC program, we believe that this issue can be managed satisfactorily. Second, field site challenges are many, including participant apprehension of ultrasound imaging, equipment malfunction and limited battery life preventing full studies, and loss of data. Some of these issues can be ameliorated by communicating effectively with study participants, securing additional batteries and/or using portable generators to maintain power to the ultrasound system and adhering to protocols to prevent data loss, for example by digitally saving the study. Third, a stable internet connection is required for uploading studies to the cloud-based system. This is not available at many of the remote locations in which studies are performed, which may require long-distance travel to secure a reliable internet connection. Fourth, difficult ultrasound scanning and/or environmental conditions may preclude optimal image acquisition. For example, BART imaging is best performed in a controlled environment with a stable, comfortable temperature and with an extended arm resting on a table, which is not always possible. Fifth, the high-quality research-grade images required for optimal measurements require practice, training and re-training on the part of sonographers. When images are considered uninterpretable, another visit is required to the study participant's home for re-imaging, which is not always possible. Finally, while these and other challenges are real, it is imperative to maintain good communication with the site sonographers, as they usually will help discover the solutions to these challenges.

## Conclusion

In conclusion, a UCL was established to ensure accurate and reproducible ultrasound measurements in clinical research. SOPs and standardized training are aimed at reducing variability and enhancing measurement precision from study sites, representing a model for the use of portable digital ultrasound images in multicenter field studies.
